# Conventional and three-dimensional photography as a tool to map distribution patterns of in-transit melanoma metastases on the lower extremity

**DOI:** 10.3389/fmed.2023.1089013

**Published:** 2023-01-19

**Authors:** Kilian Müller, Carola Berking, Caroline Voskens, Markus V. Heppt, Lucie Heinzerling, Elias A. T. Koch, Rafaela Kramer, Susanne Merkel, Beatrice Schuler-Thurner, Vera Schellerer, Theresa Steeb, Anja Wessely, Michael Erdmann

**Affiliations:** ^1^Institute of Hygiene and Environmental Medicine, University Medicine Greifswald, Greifswald, Germany; ^2^Department of Dermatology, Uniklinikum Erlangen, Deutsches Zentrum Immuntherapie (DZI), Friedrich-Alexander University Erlangen-Nürnberg (FAU), Erlangen, Germany; ^3^Comprehensive Cancer Center Erlangen–European Metropolitan Area of Nuremberg (CCC ER-EMN), Erlangen, Germany; ^4^Department of Dermatology and Allergy, University Hospital Munich, Ludwig Maximilian University of Munich, Munich, Germany; ^5^Department of Surgery, Uniklinikum Erlangen, Friedrich-Alexander University Erlangen-Nürnberg, Erlangen, Germany; ^6^Department of Pediatric Surgery, University Medicine Greifswald, Greifswald, Germany

**Keywords:** melanoma, in-transit metastasis, lower extremity, lymphatic pathways, 3D photography

## Abstract

**Background:**

In melanoma, in-transit metastases characteristically occur at the lower extremity along lymphatic vessels.

**Objectives:**

The objective of this study was to evaluate conventional or three-dimensional photography as a tool to analyze in-transit metastasis pattern of melanoma of the lower extremity. In addition, we assessed risk factors for the development of in-transit metastases in cutaneous melanoma.

**Methods:**

In this retrospective, monocentric study first we compared the clinical data of all evaluable patients with in-transit metastases of melanoma on the lower extremity (*n* = 94) with melanoma patients without recurrence of disease (*n* = 288). In addition, based on conventional (*n* = 24) and three-dimensional photography (*n* = 22), we defined the specific distribution patterns of the in-transit metastases on the lower extremity.

**Results:**

Using a multivariate analysis we identified nodular melanoma, tumor thickness, and ulceration as independent risk factors to develop in-transit metastases ITM (*n* = 94). In patients with melanoma on the lower leg (*n* = 31), in-transit metastases preferentially developed along anatomically predefined lymphatic pathways. In contrast when analyzing in-transit metastases of melanoma on the foot (*n* = 15) no clear pattern could be visualized. In addition, no difference in distance between in-transit metastases and primary melanoma on the foot compared to the lower leg was observed using three-dimensional photography (*n* = 22).

**Conclusion:**

A risk-adapted follow-up of melanoma patients to detect in-transit metastases can be applied by knowledge of the specific lymphatic drainage of the lower extremity. Our current analysis suggests a more complex lymphatic drainage of the foot.

## Introduction

Cutaneous melanoma is characterized by a high risk of metastases occurring in close proximity to the primary melanoma, along the route to and in regional lymph nodes, and as distant metastases (1). Despite approved adjuvant treatment options for primary high-risk melanoma (2) and life-prolonging therapies in the stage of distant metastasis (3), melanoma remains a fatal disease.

In-transit metastases (ITM) are a special kind of recurrence in the skin, observed predominantly in melanoma and approximately develop in 7% of the melanoma patients (4). They occur in the anatomical region between primary melanoma and the corresponding regional lymph node basin. The clinical course of patients developing ITM varies considerably, ranging from solitary lesions with prolonged relapse-free intervals up to subsequent, rapidly progressing systemic disease (1). ITM can be palpable and visible and therefore represent a significant psychological burden for patients (5). Apart from the proposed spreading along the draining lymphatic vessels, the biology of ITM formation is not fully understood and there is currently no way to identify patients at risk of developing ITM. Commonly known risk factors of melanoma including tumor thickness and ulceration of the primary melanoma also apply to patients who develop ITM. Patients with ITM at the leg have a better prognosis than those with ITM at other body sites (6). This may be related to the long lymphatic pathways of the lower extremity defined by a Japanese research group (7) in cadavers. Therefore, the area along defined lymphatic pathways where ITM occur would be predetermined based on the location of the primary melanoma.

In dermatology, different imaging modalities such as digital photography, dermoscopy, sonography, optical coherence tomography, and confocal microscopy are applied to document solitary skin lesions (8). Most of these modalities, currently predominantly applied in dermato-oncology (9) require specialized training, expensive equipment and are time consuming for the patients and medical staff (10). However, in order to image the total body, complex and standardized realignments of the camera or the patient are required. Since skin areas are distorted if they are not photographed at a vertical angle, they can only be assessed to a limited extent in conventional photography. Semiautomatic and image-processing 3D photography systems offer a patient- and user-friendly handling of total body photography (11), on which changes such as cutaneous melanoma metastases can be traced precisely using assisting image processing tools (12). These applications enable objective automated nevi count in patients with numerous pigmented lesions (13). Standardized total body 3D photography images provide a solid prerequisite for machine-based learning algorithms in dermatology (14) as demonstrated in detection and analysis of cherry angioma (15). 3D photography and customized image processing tools are applied for various dermatologic conditions: Area of vitiligo lesions was calculated accurately by manual multipoint polygon region annotation utilizing an area measurement application (16). Decrease in volume of infantile hemangioma during propranolol treatment was calculated using a portable 3D photography unit and software provided by the manufacturer (17). Wound characteristics such as area, perimeter, and volume were reliably determined by a prototype 3D-wound assessment monitor camera and for this purpose applied software (18). Facial asymmetry caused by morphea could be more properly rated by 3D stereophotogrammetry compared to 2D photography (19). Assessment of successful labial augmentation by filler injection was demonstrated using a 3D imaging system and a facial measurement and analysis tool (20). Thus, 3D images have so far been analyzed mainly by means of the software provided by the manufacturer of the camera systems. In the future, these high-resolution, standardized images, together with rapidly advancing machine learning in dermatology (21, 22), may offer great possibilities in diagnostics and management of skin diseases.

In the current work, we identified patients with ITM and melanoma of the lower extremities. To demonstrate the *in vivo* spread of tumor cells resulting in ITM along defined lymphatic pathways, standardized templates were created with annotated primary melanomas, ITM, and associated lymphatic pathways in a subset of 46 patients with available conventional and 3D photographic images.

## Materials and methods

### Patients

This monocentric, retrospective study analyzed 382 patients (female 263, male 119) with cutaneous melanoma of the lower extremity. The study was conducted in compliance with Good Clinical Practice (GCP) rules and the Declaration of Helsinki. Ethical review and approval were waived for this study due to its retrospective character and the analysis of standard patient data raised in the context of regular treatment visits. Patients signed an informed consent form for treatment at our center and for the photo documentation in the context of regular treatment. All patients underwent standard treatment and follow-up at the Department of Dermatology of the Uniklinikum Erlangen, Germany, between October 2000 and July 2020. We identified patients in our institutional database followed by individual file review from the electronic patient records in the clinical documentation system Soarian^®^ (Cerner, Health Services, Berlin, Germany). A dermatologist diagnosed ITM during clinical skin examination either at the time of primary melanoma diagnosis or during regular oncologic follow-up. Data were obtained for each patient, including gender, age at diagnosis of primary melanoma, histologic subtype (superficial spreading melanoma, nodular melanoma, acral lentiginous melanoma, melanoma not otherwise specified, or melanoma with unknown primary), mean Breslow tumor thickness (mm), ulceration (present, absent, or unknown), and localization of primary melanoma (foot, lower leg, or thigh). Additionally, we analyzed the number of detectable ITM (1–5, 6–10, 11–20, more than 20 or unknown), mean time between primary diagnosis of melanoma and first ITM (in months), and availability of clinical images (no images, conventional photography, or 3D photography).

### Image acquisition

#### Conventional photography

Images of patients were generated during follow-up at the Department of Dermatology, Uniklinikum Erlangen, Germany, by digital 2D single-lens reflex cameras (Canon EOS-ID Mark III or Canon EOS-ID Mark IV, Canon Germany GmbH, Krefeld). In the majority of the patients, primary melanoma as well as ITM had already been resected. Therefore, the location of prior melanoma lesions on clinical images by residual scars were identified and cross-referenced with the patients’ clinical file.

### 3D total body photography

Patients were imaged by a VECTRA 360WB medical photography platform system (VECTRA 360WB, Canfield Scientific, Parsippany, NJ, USA) available at our center since 2019 consisting of two concentrically orientated frameworks with a total of 92 high-resolution digital cameras that captured 92 standardized polarized images of the patient simultaneously. During image acquisition, undressed patients stood in a defined pose at a standardized position inside the imaging unit. The integrated Dermagraphix software (VECTRA-Software VAM Version 2.7.6, Canfield Scientific, Parsippany, NJ, USA) subsequently processed single images into a 3D body avatar, which covered up to 90% of the patient‘s body surface (11). Thus, a millimeter precise anatomical mapping of present lesions and scars was generated for each patient. The distance between primary melanoma and ITM was calculated *via* the skin surface distance-measuring tool of the Dermagraphix program.

### Generation of a reference template of the leg

As a first step, a reference template of the leg was created in order to compare ITM spreading pathways of the lower extremity in our patients. Contours from defined angles (lateral, medial, ventral, and dorsal) of the reference leg were delineated from an original 3D image using GIMP (GNU image manipulation program, version 2.10.20). Additionally, we integrated bone benchmarks and segmented colored lymphatic drainage areas as described previously (7): posterolateral (red), posteromedial (yellow), anterolateral (green), and anteromedial (blue—including the toes).

### Identification of melanoma lesions and transfer onto the leg template

Evaluable melanoma lesions or scars of primary melanoma and ITM were identified in 46 patients with melanoma on the lower leg or foot and cross-referenced with clinical data, previous photographic images, histologic results, and operation reports. In addition, we contacted patients in order to pinpoint the localization on conventional or 3D images. In annotated and standardized 3D images, these lesions were extracted by an affine transformation scheme consisting of 6 degrees of freedom (x, y, z, 3x rotation) and transformed back into our two-dimensional reference templates. In order to compensate individual anatomical difference between patients, images were standardized with fixed points of interest using bone benchmarks such as the patella, medial as well as lateral ankle, and toes *via* Paint-3D (Microsoft Corporation, Version 6.2004.20027.0). Conventional photographic images of melanoma lesions and scars on the leg of patients with ITM were retrieved from our photographic archive. We annotated melanoma lesions accordingly and transferred these onto the four reference templates using the software program Paint-3D (Paint-3D, Microsoft Cooperation, 6.2004.20027.0). For patients with melanoma of the right leg, annotated images were mirrored, so that a left leg was always depicted in the following analysis.

### Statistical analysis

Our objective was to evaluate conventional or three-dimensional photography as a tool to analyze in-transit metastasis pattern of melanoma of the lower extremity. In addition, we assessed risk factors for the development of in-transit metastases in cutaneous melanoma. Specifically, data were analyzed using SPSS (SPSS, Version 24.0, IBM Deutschland GmbH, Ehingen). Clinical characteristics of patients with melanoma of the lower extremity with (*n* = 94) or without ITM (*n* = 288) were compared in order to determine specific risk factors to develop ITM. The chi-square test was applied for mutually exclusive categorical variables (i.e., gender of the patient, histological subtype, ulceration, and localization of the primary melanoma). Continuous variables (i.e., age of patient, Breslow tumor thickness, and the interval between primary melanoma and the occurrence of the first ITM) were analyzed using the Mann–Whitney *U* test between two independent patient groups (patients with melanoma on the leg without ITM versus patients with melanoma of the lower leg with ITM). Multivariate logistic regression analysis of categorical as well as continuous variables was applied to factors with a significance in the univariate analysis to assess the interaction of potentially predictive factors for the development of ITM of the lower extremity. In order to analyze differences between fully (*n* = 25), partially (*n* = 8), or non-matched (*n* = 15) metastatic patterns of ITM between the lower leg (*n* = 31) and the foot (*n* = 15) we applied the Mann–Whitney *U* test of ordinally scaled variables in the two independent sample groups (melanoma on the leg versus melanoma on the foot). In all tests, a *p*-value < 0.05 was considered significant.

## Results

### Clinical risk factors of all patients with in-transit metastases on the lower extremity

Demographic and clinical characteristics were compared between 288 patients with melanoma on the lower extremity without ITM and 94 patients with melanoma on the lower extremity with ITM. Patients with ITM were significantly older and the primary melanoma was significantly more often a nodular or acral lentiginous subtype, had a greater tumor thickness, showed more commonly ulceration, and was located more distally compared to patients without ITM (*p* < 0.001) (Table 1). In a multivariate logistic regression analysis (Table 2) tumor thickness, ulceration, and nodular melanoma remained risk factors for development of ITM. Independent of the presence of ITM, in our cohort melanoma on the lower extremity was more common in women. In the 94 patients, the number of ITM ranged from a single lesion to more than 20 lesions. The time between diagnosis of primary melanoma and of ITM ranged from simultaneous detection to more than 20 years (Table 1).

**TABLE 1:** General patient characteristics.

		*N*	Patients without ITM *n* = 288	Patients with ITM *n* = 94	*P*-value
Gender	Male	119	86 (30%)	33 (35%)	0.340
	Female	263	202 (70%)	61 (65%)	
	Male-to-female ratio		1:2.4	1:1.9	
Age (years)	Mean		53	62	<0.001
	Median		53	65	
	Range		16–97	28–96	
Histologic subtype	SSM	226	198 (69%)	28 (30%)	<0.001
	NM	62	38 (13%)	24 (26%)	
	ALM	43	25 (9%)	18 (18%)	
	NOS	35	27 (22%)	21 (22%)	
	MUP	3	–	3 (3%)	
Tumor thickness (mm)	Mean		1.4	3.5	<0.001
	Median		0.9	1.9	
	Range		0.1–35.0	0.2–15.0	
	Unknown		–	8 (8%)	
Ulceration*	Present	81	44 (15%)	37 (46%)	<0.001
	Absent	288	244 (85%)	44 (54%)	
Location of melanoma**	Foot	75	49 (17%)	26 (28%)	<0.001
	Lower leg	159	112 (39%)	47 (52%)	
	Thigh	145	127 (44%)	18 (20%)	
Number of ITM	1–5			34 (36%)	
	6–10			18 (19%)	
	11–20			14 (15%)	
	>20			15 (16%)	
	Unknown			13 (14%)	
ITM localization**	Only proximal of melanoma			46 (80%)	
	Distal and proximal of melanoma			9 (20%)	
Time between melanoma and first ITM [months]	Mean			34	
	Median			19	
	Range			0–244	
Images (including MUP)	3D photography			29 (31%)	
	Conventional photography			32 (34%)	
	No evaluable images available			33 (35%)	

ITM, in-transit metastasis; SSM, superficial spreading melanoma; NM, nodular melanoma; ALM, acral lentiginous melanoma; NOS, not otherwise specified; MUP, melanoma of unknown primary; 3D, three-dimensional.

*Ulceration status was unknown (*n* = 10) or a MUP was present (*n* = 3).

**MUP (*n* = 3). Characteristics of patients with melanoma of the lower extremity with in-transit metastases (*n* = 94) compared to patients without recurrence of melanoma (*n* = 288) with regard to primary melanoma and in-transit metastases.

**TABLE 2:** Multivariate logistic regression analysis of risk factors for in-transit metastases of melanoma of the lower extremity.

		Univariate analysis			Multivariate analysis		
		**Hazard ratio**	**95% CI**	***P*** **-value**	**Hazard ratio**	**95% CI**	***P*** **-value**
Age (years)	<55	1.0			1.0		
	≥55	2.5	1.4–4.3	**<0.001**	1.5	0.8–2.8	0.214
Histologic subtype*	SSM	1.0			1.0		
	NM	4.7	2.4–9.0	**<0.001**	2.6	1.2–5.5	**0.011**
	ALM	4.6	2.1–9.8	**<0.001**	2.7	0.8–9.2	0.121
	NOS	2.1	0.8–5.4	0.111	1.7	0.6–4.9	0.296
Tumor thickness (mm)*	<1.0	1.0			1.0		
	≥1.0	10.5	4.6–23.6	<0.001	5.7	2.3–13.9	**<0.001**
Ulceration*	Present	1.00			1.0		
	Absent	5.1	2.9–8.9	**<0.001**	2.1	1.1–4.0	**0.032**
Location of melanoma	Foot	1.0			1.0		
	Lower leg	0.8	0.4–1.4	0.374	1.8	0.6–5.0	0.272
	Thigh	0.3	0.1–0.6	**<0.001**	0.6	0.2–1.8	0.349

CI, confidence interval; SSM, superficial spreading melanoma; NM, nodular melanoma; ALM, acral lentiginous melanoma; NOS, not otherwise specified; MUP, melanoma of unknown primary.

*Missing values: histologic subtype *n* = 17, tumor thickness *n* = 8, ulceration *n* = 13. Multivariate logistic regression analysis of significant variables in the univariate analysis was performed in a full dataset of 359 patients with melanoma of the lower extremity.

Bold values represent the *p* < 0.05.

### Analysis of metastatic distribution pattern of in-transit metastases along lymphatic drainage pathways in 46 patients with evaluable conventional and three-dimensional photography

**FIGURE 1:**
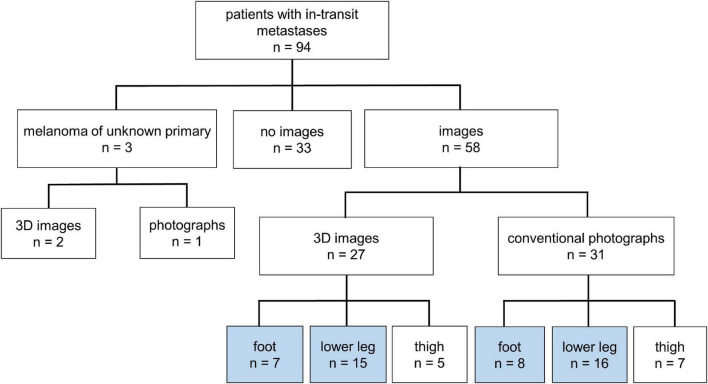
Overview of melanoma patients with in-transit metastases (*n* = 94) of the lower extremity. Definition of patients with available images taken by conventional or three-dimensional (3D) photography of the upper leg, lower leg, and foot.

**FIGURE 2:**
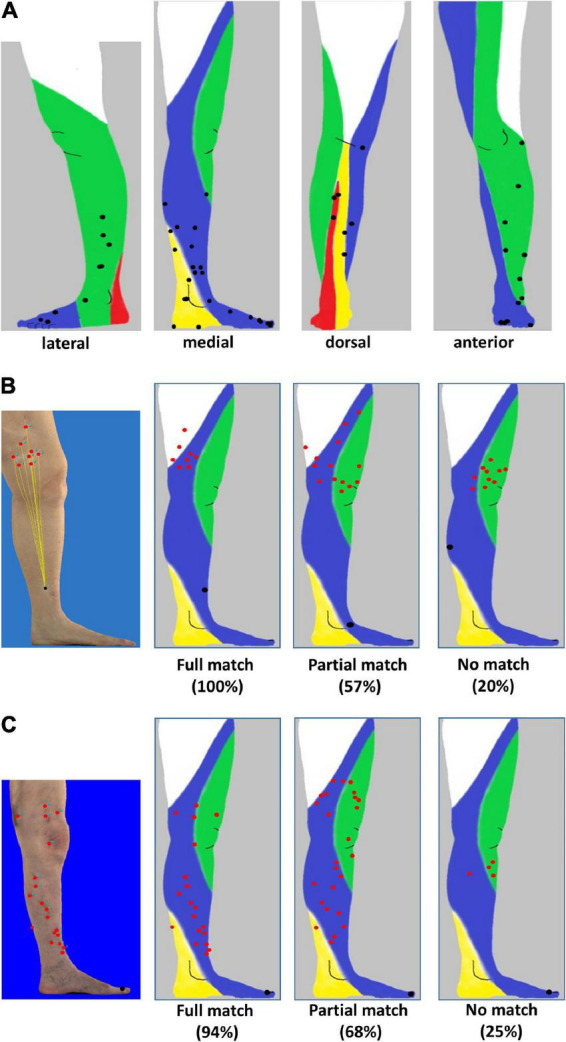
Distribution of primary melanoma and in-transit metastases localized to the lower leg and foot. **(A)** Lateral, medial, anterior, and dorsal projection of primary melanoma (black) of 58 patients based on photographic images and mapping to distinct anatomic lymphatic drainages regions of leg: posteromedial (yellow), anteromedial (blue), anterolateral (green), and posterolateral (red). **(B)** Melanoma of the lower leg: three-dimensional (3D) image of a primary melanoma and corresponding in-transit metastases as well as the supposed lymphatic drainage (left image). Representative examples of individual patients with primary melanoma (black) on the lower leg with a full match of 8/8 (100%) of in-transit metastases (red) developing in the corresponding anteromedial lymphatic region (second image from the left) and metastases developing partially (8/14, 57%, second image from the right) or not predominantly within (2/10, 20%) the expected localization (right image). **(C)** Melanoma on the foot. Annotation of in-transit metastases (red) on a 3D photographic image (left image). Examples of representative individual patients with primary melanoma (black) and corresponding in-transit metastases (red) occurring fully, partially, or not (images second from the left, second from the right, and right) in the expected lymphatic drainage area.

A total of 46 patients with melanoma of the lower leg (*n* = 31) or the foot (*n* = 15) and evaluable conventional (*n* = 24) or 3D-photography (*n* = 22) could be identified in our photo database (Figure 1, blue boxes) to correlate individual localization of ITM with previously reported anatomic lymphatic drainage pathways of the lower leg (7). Clinical data of these patients are summarized in Table 3. The anatomical distribution of all primary melanomas on the lower extremity and foot is shown in Figure 2A. We detected a strong correlation to known lymphatic vessels (full match) between primary melanoma of the lower leg (*n* = 31) and corresponding ITM (representative examples in Figure 2B) along the anteromedial (blue) or the anterolateral (green) lymphatic draining pathway in 22 of 31 patients (71%) (Table 4). A partial match was demonstrated in two patients (6%). In seven patients (23%), no association between the localization of primary melanoma and ITM could be detected. In melanoma of the foot (*n* = 15), a full and partial match between primary melanoma and ITM along the lymphatic pathways was present in only three patients (20%) and six patients (40%), respectively (representative examples in Figure 2C and Table 4). No congruence was detected in six patients (40%).

**TABLE 3:** Patients with melanoma of the lower leg and foot with analyzed in-transit metastases patterns.

		*n*	Conventional photography *n* = 24	3D-photography *n* = 22
Gender	Male	10	5 (21%)	5 (23%)
	Female	36	19 (79%)	17 (77%)
	Male-to-female ratio		1:3.8	1:3.4
Age (years)	Median		66	60
	Mean		70	66
	Range		28–87	29–87
Histologic subtype	SSM	12	7 (29%)	5 (23%)
	NM	9	6 (25%)	3 (14%)
	ALM	11	5 (21%)	6 (27%)
	NOS	14	6 (25%)	8 (36%)
Tumor thickness (mm)*	Median		3.5	3.3
	Mean		3.6	3.0
	Range		0.2–7.3	0.3–7.3
	Unknown		1 (4%)	2 (9%)
Ulceration*	Present	21	15 (79%)	6 (30%)
	Absent	18	4 (21%)	14 (70%)
Location of melanoma	Foot	15	8 (33%)	7 (32%)
	Lower leg	31	16 (67%)	15 (68%)
Number of ITM	1–5	13	4 (17%)	8 (36%)
	6–10	12	6 (25%)	7 (32%)
	11–20	11	7 (29%)	4 (18%)
	>20	10	7 (29%)	3 (14%)
Time between melanoma and first ITM (months)	Median		8	31
	Mean		22	53
	Range		0–120	0–244

ITM, in-transit metastasis; SSM, superficial spreading melanoma; NM, nodular melanoma; ALM, acral lentiginous melanoma; LMM, lentigo maligna melanoma; NOS, not otherwise specified; 3D, three-dimensional.

*Ulceration status was unknown (*n* = 7). Patient characteristics of 46 patients with primary melanoma of the lower extremity and the foot with in-transit metastases were analyzed by conventional or 3D photography.

**TABLE 4:** Classification of in-transit metastatic patterns defined by localization of primary melanoma.

	Foot (*n* = 15)	Lower leg (*n* = 31)	*P*-value
Full match	3 (20%)	22 (71%)	
Partial match	6 (40%)	2 (6%)	0.007
No match	6 (40%)	7 (23%)	

Depending on the occurrence of in-transit metastases in the corresponding anatomical area of the primary melanoma, metastatic patterns are classified as full match (≥90%), partial match (<90% and ≥50%), or no match (<50%). While in melanoma of the lower leg the majority of in-transit metastases develop in the corresponding lymphatic drainage areas, melanoma of the foot shows a different metastatic pattern.

### Absolute distance between primary melanoma and ITM measured *via* three-dimensional photography

**FIGURE 3:**
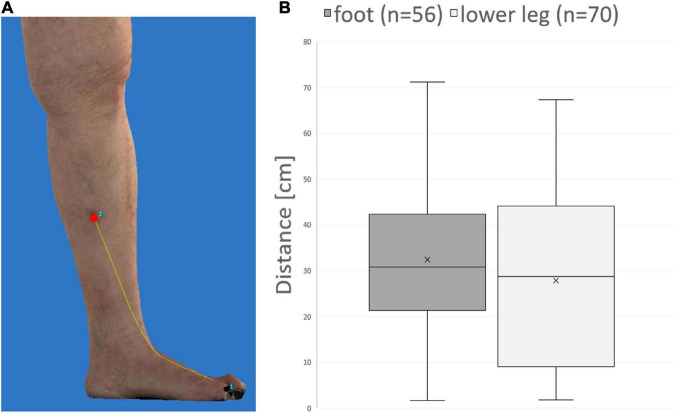
Distance measurement of primary melanoma and in-transit metastases on three-dimensional (3D) photography. **(A)** Example demonstrating measurement of the distance (yellow line) along the skin surface between primary melanoma (black) and an in-transit metastasis (red). **(B)** No significant difference between melanoma of the foot compared to the lower leg is detectable concerning mean, median, and range of distances between melanoma and corresponding in-transit metastases.

Using 3D photography, the distance between primary melanoma and ITM along the skin surface could be measured in 27 patients (Figure 3A). Anatomically, the distance between primary melanoma and draining lymph nodes in the groin increases from the foot compared to the lower leg. Nevertheless, the mean, median, and range of distance between primary melanoma on the foot (27.8, 28.8, 1.8–67.4 cm) or lower leg (32.5, 30.9, and 1.6–71.2 cm) and corresponding ITM showed no significant difference in our cohort (Figure 3B).

## Discussion

In-transit metastases develop along the way between the primary melanoma and the draining nodal basin and are a poor prognostic sign (6). Currently, the etiology of ITM is not fully understood. In the present study, conventional and 3D photography were used to chart and analyze the metastatic distribution of ITM of primary cutaneous melanoma of the lower extremity for the first time with regard to anatomically determined lymphatic drainage pathways (7) in a subset of 46 patients.

In general, about 4 to 10% of melanoma patients develop ITM (6, 23). The high rate of ITM (33%) in our clinical patient collective of 382 patients might be explained by the fact that it was limited to melanoma of the lower extremity, where ITM more often occur compared to other body sites (6). Additionally, patients with advanced or metastatic melanoma are more likely referred to a university skin cancer center, resulting in a high number of patients with ITM in our analysis.

Our clinical cohort of 94 patients with ITM complements reports that comprised 54 (24), 380 (25), and 505 patients (6) with satellite metastases and ITM. While in our study only patients with melanoma of the lower extremity were included, this subgroup makes 40–60% of the comparable collectives in the literature. In particular, the different biology and prognosis of acral lentiginous melanoma (26) represent a possible confounder. The median tumor thickness of 2.9 mm and ulceration status of 46% in our patient cohort were comparable with other studies reporting a median tumor thickness of 3 mm and the presence of ulceration in 28–39% of the patients (6, 24). Tumor subtypes and further localization on the thigh, lower leg, or foot could not be compared because they were not stated separately for the lower extremity (6) or were not reported at all (24, 25). With regard to patient characteristics, the male-to-female ratio ranged from 1:2 in our study to 2:1 in the literature (6). This different distribution may be influenced by differences in the country-specific risk of sun exposure and behavior between Europe and Australia (27). The median age of patients was 65–75 years and confirms that ITM occur at an older age, but could not be exactly compared due to different timing at data collection (primary melanoma diagnosis or start of systemic therapy). In our study, the median interval until occurrence of ITM was 19 months, which is in line with 17.9 months stated in the literature (6). The number of ITM per patient has not been reported in the literature. The gender distribution in our patients with melanoma on the lower extremity was independent of the occurrence of ITM. Our data confirm negative prognostic factors in melanoma (1, 28) in patients with ITM compared to patients without recurrence such as tumor thickness, ulceration status, and nodular melanoma.

In a subset of 46 patients with evaluable conventional and 3D photography, the localization of melanoma lesions was systematically correlated to anatomically determined lymphatic drainage pathways (7). Our results show that the localization of primary melanoma on the lower leg indicates areas at risk which should be examined more closely during follow-up. In contrast, a full match between ITM and lymphatic drainage was present in only 20% of the patients with melanoma of the foot. This discrepancy may be caused by altered lymphatic drainage pathways due to surgery of the toes and sole of the foot. The fact that 9 of our 46 analyzed patients developed ITM distally of the primary melanoma indicates a significant surgical alteration of the lymphatic vessels including the draining nodal basin by prior sentinel node biopsy or lymphadenectomy.

Interestingly, one patient with amputation of the three toes due to melanoma developed ITM along both the anteromedial and the anterolateral lymphatic bundles. This may further support postoperative lymphatic alteration but could also indicate a yet unstudied differential lymphatic drainage of the toes and the plantar region. It is of interest that in order to identify anatomic lymphatic pathways, contrast injections were exclusively applied to the outer and inner edges of the foot as well as the interdigital spaces in a previous study (7).

As discussed by Shinaoka et al. (7), the results of the anatomic/radiologic study may not be fully applicable to the *in vivo* situation (such as the development of ITM analyzed in this work) as the study was performed on cadavers with contrast injection into the foot followed by manual lymphatic drainage. In contrast, *in vivo* lymphatic fluid is transported by means of metabolically active muscle contraction in conduit and transport vessels.

To our knowledge, this is the first report to apply 3D photography to determine precise distances along the lymphatic pathways that melanoma cells must have traveled between primary melanoma and corresponding ITM. Since the distances between two lesions were calculated on a 3D leg model on the skin surface and the angles of the joints were taken into account, this almost exactly corresponds to the lymphatic pathways running in the upper corium. Although lymphatic pathways from the foot to the draining inguinal lymph node are anatomically longer than those from the lower leg, the distances between primary melanoma and associated ITM did not differ significantly in our cohort. Whether this is a biological, mechanical, or lymphogenic characteristic of melanoma of the foot, in which ITM develops with preferentially on the lower leg and thigh, cannot be answered by this study due to the insufficient number of patients examined by 3D photography (*n* = 22). In order to assess the significance for diagnostics and therapy, further investigations of ITM should also be performed including the trunk, the heck, and head.

Our study demonstrates for the first time the biological relevance of anatomically described lymphatic drainage pathways using ITM in melanoma of the lower extremity as a model. However, the limitations of our work must be considered before this knowledge can be applied for example to assess risk areas for ITM: (i) In our clinical collective we could only identify 94 patients with ITM of the leg in our center within a 20-year time frame, (ii) the analysis of ITM along lymphatic vessels was limited to melanoma of the lower leg and foot as only in the distal lower extremity lymphatic drainage patterns have been systematically investigated, (iii) evaluable photo documentation to analyze the lymphatic drainage patterns of melanoma of the lower leg and foot was only available in 46 patients, (iv) analysis of conventional, non-standardized photographs proved to be particularly difficult and possibly error-prone compared to 3D total body photography (in use in our department since 2019), (v) the localization of melanoma lesions was determined almost exclusively based on the presence of scars and comparison of the patients’ medical records. Especially for primary melanomas excised with a safety margin (sometimes toe amputation), the localization of melanoma lesions had to be extrapolated in the middle of the scar, (vi) changes in the physiologic lymphatic drainage pathways due to surgery around primary melanomas, ITM, and lymphadenectomy may have occurred in all patients, and (vii) due to methodological reasons, the anatomical study did not examine toe and plantar drainage, which remain not well defined. Therefore, the poor correlation observed between ITM and primary melanoma at the foot in our cohort suggests a critical re-evaluation with regard to lymphatic drainage pathways of the toes and the plantar region.

In summary, our results demonstrate the *in vivo* relevance of anatomic lymphatic pathways in ITM formation in 46 evaluable patients with melanoma of the leg. Together with clinical risk factors that we have confirmed, this knowledge may be used for a better risk assessment in the follow-up of melanoma patients and to develop a model for ITM in melanoma of the leg in the future.

## Data availability statement

The raw data supporting the conclusions of this article will be made available by the authors, without undue reservation.

## Author contributions

ME and KM designed the study, performed data acquisition and analysis, and wrote the manuscript. ME, KM, and SM interpreted the data. CB, CV, MH, LH, EK, RK, and BS-T collected patient data and clinical information. CB, CV, MH, LH, EK, RK, SM, BS-T, VS, TS, and AW critically revised the manuscript. All authors contributed to the article and approved the submitted version.
